# A registration method for total hip arthroplasty navigation system based on point cloud alignment

**DOI:** 10.3389/fmedt.2026.1775840

**Published:** 2026-05-15

**Authors:** Zhenling Wang, Qiurui He, Xinwei Yue

**Affiliations:** 1 The School of Internet of Things Engineering, Wuxi University of Technology, Wuxi, China; 2 The School of Software, Luoyang Normal University, Luoyang, China; 3 The School of Information and Communication Engineering, Beijing Information Science and Technology University (BISTU), Beijing, China

**Keywords:** ICP algorithm, image point cloud sampling, patient registration, sparse-dense point cloud alignment, surgical navigation system

## Abstract

Hip replacement surgery requires high precision in aligning intraoperative anatomical structure localization with preoperative imaging spatial alignment. To address the registration difficulties caused by the limited number and sparse distribution of intraoperative probe acquisition points, our paper proposes a method based on point cloud registration between an intraoperative probe tracked by a passive binocular optical tracking system (NDI) and a CT surface, aimed at improving patient registration accuracy in intraoperative navigation scenarios. First, we define the patient-registration problem and describe the methods for acquiring pre-operative and intra-operative data. Subsequently, the centroids of the NDI optical tracking markers are employed as common features shared by the patient and image spaces, and an initial registration is performed using a three-point method. Finally, an improved ICP algorithm is used to perform accurate registration between the two point clouds, and the registration results are validated using a fixture platform. In the experiments conducted on 3D-printed acetabular and femoral models, the target registration errors (TRE) of the proposed method for registering the acetabular model and the femoral model are (0.55 ± 0.15) mm and (0.67 ± 0.18) mm, respectively. The experimental results demonstrate that the proposed method achieves good registration accuracy and stability under phantom experimental conditions. Using the TRE as the primary evaluation metric to measure the spatial localization error of target points, the method exhibits superior geometric registration performance compared to the traditional iterative closest point (ICP) algorithm. Additionally, it features a concise workflow and ease of implementation, providing a feasible technical solution for sparse point cloud registration in intraoperative navigation scenarios.

## 1 Introduction

The integration of computer technology, medical image processing, and surgical navigation techniques has driven the development of digital orthopedic surgery [1]. Surgical navigation systems can provide surgeons with spatial reference information regarding anatomical structures and instrument positions during surgery, thereby improving the consistency and visualization level of surgical operations. Patient registration is a key technique in surgical navigation systems, aiming to obtain the rigid transformation between the physical space of the patient and the image space where the three-dimensional virtual model resides, thereby achieving consistency between the preoperative imaging data and the intraoperative spatial position of the patient. The accuracy and efficiency of registration largely determine the overall performance of the navigation system.

In recent years, surgical navigation technology has been extensively studied in Total Hip Arthroplasty (THA) [2–7]. Related studies have shown that navigation systems can effectively improve the consistency and reproducibility of acetabular cup positioning during surgery. With the advancement of navigation technology, various techniques such as CT-based navigation, imageless navigation, and augmented reality navigation have been gradually applied in THA surgery. For example, Hasegawa et al. developed a CT-based augmented reality navigation system that overlays imaging information onto the patient’s anatomy, providing surgeons with intuitive spatial guidance and thereby improving the accuracy of acetabular cup positioning during surgery [3]. Additionally, some studies have proposed surgical navigation systems based on inertial sensors or imageless navigation to achieve more convenient and cost-effective intraoperative navigation [5], which can also significantly improve surgical positioning accuracy. However, in these navigation systems, achieving high-precision spatial registration between the preoperative CT model and the intraoperative patient anatomy remains one of the core technical challenges.

Patient registration is accomplished by identifying at least three identical fiducial points described in both the image space and the patient space [8], after which a registration algorithm computes the transformation. Traditional fiducial-based approaches typically involve implanting medical markers into the patient’s bone or affixing them to the skin surface, achieving a registration accuracy of approximately 0.5–1.5 mm [9, 10]. Although this method offers high accuracy, it may increase operational complexity in practical applications and impose additional burdens on the patient. Lin et al. developed a real-time, automatic patient-registration method based on an optical tracking system, achieving a mean registration error of about 0.7 mm [11]. However, when this method is used, if the markers become displaced or the reconstructed model is defective, the registration error may increase significantly.

With the rapid advancement of three-dimensional scanning and reconstruction technologies, point-cloud data acquisition and processing have become increasingly mature. Owing to advantages such as small data size, rapid processing, and retained structural information, point-cloud processing has found broad application in healthcare and other fields [12]. Numerous studies on 3D point-cloud registration have already been conducted worldwide. Koide et al. [13] proposed the Voxelized Generalized Iterative Closest Point (VGICP) algorithm for fast and accurate three-dimensional point-cloud registration. The method extends the Generalized Iterative Closest Point (GICP) algorithm by approximating the point-cloud geometry with a uniformly sized 3D voxel grid, thereby avoiding costly nearest-neighbor searches while preserving accuracy. Le et al. developed a robust, correspondence-free stochastic algorithm for point-cloud registration that solves 3D alignment via graph matching and semidefinite relaxation [14]. Yao et al. [15] proposed an enhanced Iterative Closest Point algorithm that leverages curvature-feature similarity of point clouds to improve the accuracy, robustness, and stability of the classical ICP in unstructured environments. Shi et al. [16] introduced a KD-tree-enhanced ICP algorithm that combines point-cloud filtering with a bioinspired stochastic search algorithm [17] for coarse registration. The method augments the conventional KD-tree-enhanced ICP algorithm with an additional point-cloud filtering stage and an adaptive fireworks coarse-registration step. He Y et al. [18] proposed an ICP registration algorithm based on local point-cloud features. First, leveraging the point cloud’s intrinsic feature information, they design robust and efficient 3D local feature descriptors—density, curvature, and normal angle. Based on these feature descriptors, correspondences between the point clouds are established and an initial registration result is obtained. Finally, the obtained result is supplied as the initial pose to ICP, enabling fine-tuning of the registration. Liu et al. [19] developed an improved registration algorithm based on Simulated Annealing (SA) and Markov Chain Monte Carlo (MCMC) theory to attain the global minimum from any initial condition. In recent years, deep learning methods have also been gradually introduced into surgical navigation registration. Some studies have combined neural networks with point cloud registration algorithms to improve the accuracy and robustness of spatial registration [20–29]. For example, Liu et al. proposed a hybrid framework combining neural networks with point cloud registration for spatial registration in augmented reality neurosurgical navigation, thereby improving registration accuracy [26]. The authors systematically summarized the feature extraction mechanisms of different deep learning models in LDCT denoising [29]. The study points out that traditional methods rely on manually designed filters, whereas deep learning methods can automatically extract hierarchical features through convolutional neural networks (CNNs), enabling modeling from noise distribution to structural information. In [22], the authors proposed a dual-stage deep learning framework, the core of which lies in the feature extraction and fusion of multimodal medical images. This method leverages the complementary information among different MRI modalities (such as T1, T2, and FLAIR) and extracts multi-channel features through deep convolutional networks. The study emphasizes that effective feature extraction not only requires capturing global information but also enhancing the discriminative capacity of local lesion regions. In [20], the authors classified radiological images based on a CNN model, with the core focus on learning discriminative features to distinguish different lung lesions. In this study, the CNN progressively extracts features through multiple convolutional operations, and the model maps features to the class space via fully connected layers, thereby completing the pneumonia type classification task.

In summary, deep learning methods offer significant advantages in feature extraction capabilities, but their ”data-driven” nature also brings challenges such as limited interpretability and poor engineering controllability. Particularly in tasks involving spatial geometric relationship modeling, relying solely on deep features often makes it difficult to ensure the stability and consistency of the results. In the context of sparse point cloud registration, traditional methods are based on strict geometric constraints and optimization theory, achieving the solution of registration parameters by explicitly modeling the spatial relationships between point clouds. Their feature extraction process—such as local geometric features, normal vectors, and correspondences—has clear physical meaning, endowing the entire registration process with good interpretability. Moreover, these methods are less dependent on data scale, feature a clear algorithmic structure, and better meet the requirements of engineering systems for stability and reliability. Therefore, traditional point cloud registration methods continue to hold significant value in practical applications. This paper uniformly adopts the TRE as the core evaluation metric, supplemented by the RMSE, STD, and maximum error for comprehensive assessment.

### 1.1 Motivation and contributions

Although many ICP variants (such as point-to-plane ICP, GICP, SparseICP, and robust ICP) have been proposed to improve registration accuracy and robustness, these methods are primarily designed for dense point cloud registration scenarios. However, in surgical navigation systems, data acquired by intraoperative probes typically contains only a small number of sparse surface points. Direct application of traditional ICP variants may lead to unstable correspondences, thereby reducing registration accuracy. Therefore, this study focuses on the registration problem between sparse intraoperative probe points and CT surface models. Combining the application requirements of surgical navigation systems and the characteristics of ICP algorithms, with the important goals of improving registration accuracy and operability, we propose a method based on intraoperative NDI probe and CT surface point cloud registration to enhance the precision of surgical navigation systems. Therefore, our study focuses on the registration problem between sparse intraoperative probe points and CT surface models. Combining the application requirements of surgical navigation systems and the characteristics of ICP algorithms, with the important goals of improving registration accuracy and operability, we propose a method based on intraoperative NDI probe and CT surface point cloud registration to enhance the precision of surgical navigation systems. Unlike deep learning-based registration methods, the proposed method specifically targets sparse point cloud data acquired by intraoperative probes and improves upon traditional registration methods. Compared with deep learning methods that rely on large amounts of training data and have limited interpretability, traditional methods based on explicit geometric constraints still hold significant research value.

It should be noted that the research in this paper is conducted based on 3D-printed phantom experiments and does not involve direct comparison with clinical navigation systems or surgical robots. The main contributions of our study are as follows:

1. To address the problem of insufficient registration accuracy between intraoperative anatomy and preoperative CT models in Total Hip Arthroplasty (THA) navigation systems, we propose a point cloud registration method based on the NDI optical tracking system to achieve high-precision alignment between preoperative images and intraoperative space.
2. Aiming at the characteristics of limited quantity and sparse distribution of points collected by intraoperative probes, we improve the traditional SparseICP algorithm: extending it to point-to-plane registration to improve the convergence speed and accuracy of sparse point clouds, and introducing a coarse registration stage to provide initial poses for fine registration. Through two-stage registration (coarse registration + local sparse point-to-plane ICP), high-precision registration between intraoperative local sparse point clouds and CT surface point clouds is achieved. This method is clearly distinguished from traditional global dense point cloud registration methods such as ICP, GICP, and SparseICP, and the proposed method is more suitable for addressing the problem of intraoperative local sparse point cloud registration, thereby demonstrating its methodological specificity for this particular application scenario.
3. An experimental validation platform based on pelvic and femoral models and an optical tracking system was constructed: using 3D printed models and CT scan data to extract point clouds, the effectiveness of the proposed method in terms of registration accuracy, robustness, and repeatability is validated through multiple sets of experiments. The experimental results show that the spatial positioning errors of the proposed method in femoral and acetabular registration are 0.67 ± 0.18 mm and 0.55 ± 0.15 mm, respectively, which are significantly better than those of the traditional ICP algorithm and its improved variants.

### 1.2 Organization

The rest of this paper is organized as follows. In Section 2, the problem formulation and data acquisition for patient registration is presented. In Section 3, the registration method is investigated. Furthermore, a case study in registration experiments is presented in detail in Section 4. Finally, Section 5 concludes the paper.

## 2 Problem formulation and data acquisition for patient registration

**Figure 1:**
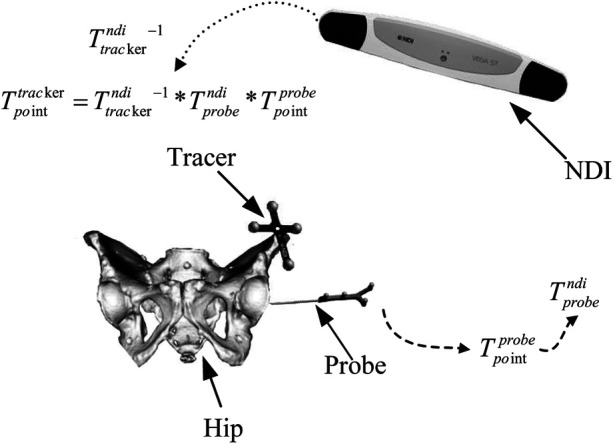
Surgical navigation system components and coordinate transformations.

A surgical navigation system is typically divided into three subsystems: the optical tracking subsystem, the image subsystem, and the patient subsystem. The optical positioning subsystem adopts the Polaris Vega passive optical 3D localizer (NDI, Canada). Its working components include a position sensor and passive localization tools—a probe pointer and rigid-body trackers. Each tracker is fitted with four reflective markers that are coplanar yet non-collinear. The position sensor tracks and outputs either the centroid coordinates of every marker or the pose of the tracker’s overall reference point. In the patient subsystem, the acetabulum serves as the treatment target; a rigid-body tracker is rigidly fixed to the acetabulum to coordinate intra-operative navigation. The image subsystem processes and visualizes the patient’s CT data and 3D model; the coordinate transformations among all subsystems are shown in Figure 1.

The transformation matrices among the subsystems’ coordinate frames are derived as follows:

1. $T_{\mathrm{probe}}^{ndi}$ and $T_{\mathrm{tracker}}^{ndi}$ represent the real-time spatial relationship between the probe and the tracker in the NDI coordinate frame, acquired live by the NDI system.
2. $T_{\mathrm{point}}^{\;probe}$ denotes the position of the probe tip in the probe’s own coordinate frame; the transformation matrix T can be determined either by three-coordinate calibration or via an NDI calibrator, and it represents a fixed, constant relationship.
3. During surgery, the tracker is rigidly fixed relative to the patient’s bone, maintaining an unchanging positional relationship. To ensure experimental accuracy, the probe-tip position must be expressed in the tracker coordinate frame. The transformation is given as Equation 1:
  $$T_{\mathrm{point}}^{tracker}=(T_{\mathrm{tracker}}^{ndi}{)}^{-1}*T_{\mathrm{probe}}^{ndi}*T_{\mathrm{point}}^{\;probe}.$$ (1)
  The surgical navigation system aims to determine the transformation that maps point $P_{\mathrm{ndi}}$ in the tracker coordinate system to point $P_{\mathrm{ct}}$ in the CT coordinate system, which expressed as Equation 2:
  $$P_{\mathrm{ct}}=T_{\mathrm{precise}}*T_{\mathrm{coarse}}*P_{\mathrm{ndi}},$$ (2)
  where $T_{\mathrm{coarse}}$ is the coarse-registration matrix, and $T_{\mathrm{precise}}$ is the fine-registration matrix.

Matrix $T_{\mathrm{precise}}$ is computed using a point-cloud registration algorithm. In an orthopedic surgical navigation system, the necessary pre-operative and intra-operative point-cloud data are acquired as follows:

1. The NDI tracker is rigidly fixed to the patient’s bone and CT-scanned together with it. Next, the CT data are imported into 3D Slicer to reconstruct a 3D model, thereby defining the image space I. A complete surface point cloud $M_{I}$ is then extracted from the bone model, typically comprising tens to hundreds of thousands of points.
2. In the patient’s physical space P, the probe tip is used to touch the exposed bone surface several times, acquiring a point cloud $V_{p}$ that typically contains only a few dozen samples. This is due to the limited surgically accessible area imposed by the bone’s anatomical structure. Consequently, patient registration is reduced to aligning a dense target point cloud $M_{I}$ (in image space) with a sparse source point cloud $M_{P}$ (in patient space). These two sets differ dramatically in sample count and exhibit pronounced disparities in geometric characteristics such as contour and shape.

## 3 Registration method

### 3.1 Initial registration based on the three-point method

$P_{\mathrm{ct}}$ and $P_{\mathrm{ndi}}$ denote the coordinates of the points in the CT and NDI coordinate systems, respectively. R is the rotation matrix between the two coordinate systems. T is the translation vector from the NDI coordinate system to the CT coordinate system. The relationship between $P_{\mathrm{ct}}$ and $P_{\mathrm{ndi}}$ is given by Equation 3:

$$P_{\mathrm{ct}}=R*P_{\mathrm{ndi}}+T.$$ (3)

**Figure 2:**
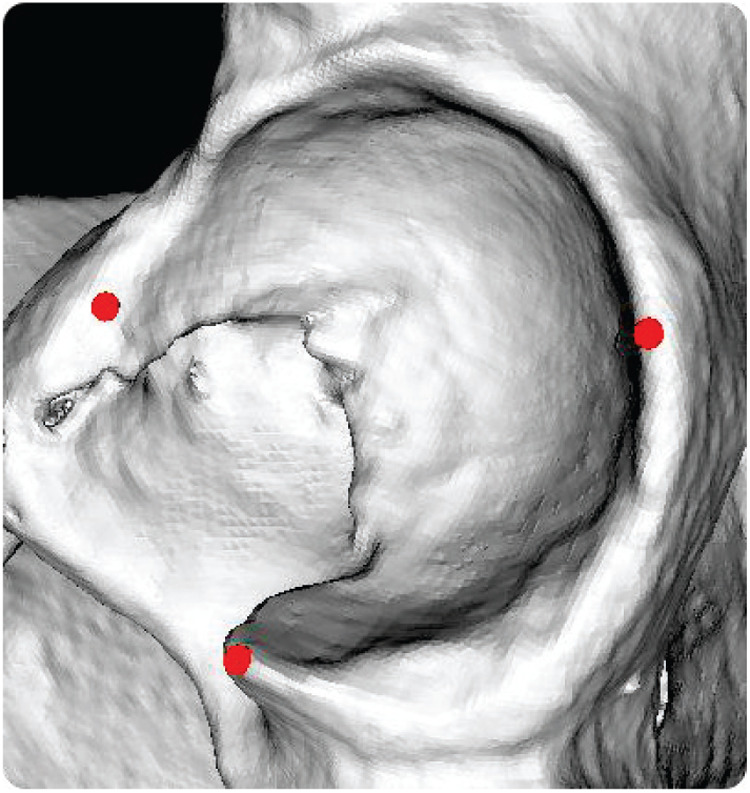
Three-point coarse registration.

To obtain the initial registration matrices for R and T, three point pairs are manually selected. As shown in Figure 2, points $P_{\mathrm{CT}}^{i}=[x_{\mathrm{CT}}^{i},y_{\mathrm{CT}}^{i},z_{\mathrm{CT}}^{i}{]}^{T}$ are marked on the acetabulum in the CT image, while the corresponding points $P_{\mathrm{NDI}}^{i}=[x_{\mathrm{NDI}}^{i},y_{\mathrm{NDI}}^{i},z_{\mathrm{NDI}}^{i}{]}^{T}$ are located on the patient’s bone surface at the same anatomical positions using a tracked probe. The initial registration matrices for *R* and *T* are computed as follows Equations 4–9:

$$H=\sum_{i=1}^{N}(P_{\mathrm{NDI}}^{i}-{\text{centroid}}_{\mathrm{NDI}}){(P_{\mathrm{CT}}^{i}-{\text{centroid}}_{\mathrm{CT}})}^{T},$$ (4)

$${\mathrm{centroid}}_{\mathrm{NDI}}=\frac{1}{N}\sum_{i=1}^{N}P_{\mathrm{NDI}}^{i},$$ (5)

$${\mathrm{centroid}}_{\mathrm{CT}}=\frac{1}{N}\sum_{i=1}^{N}P_{\mathrm{CT}}^{i},$$ (6)

[U,S,V]=SVD(H), (7)

$$R=VU^{T},$$ (8)

$$T=-R*{\mathrm{centroid}}_{\mathrm{NDI}}+{\mathrm{centroid}}_{\mathrm{CT}},$$ (9)

Where ${\mathrm{centroid}}_{\mathrm{NDI}}$ and ${\mathrm{centroid}}_{\mathrm{CT}}$ denote the mean centers in the CT and NDI coordinate systems, respectively; H is the covariance matrix, and $T_{\mathrm{NDI}}^{CT}=[R,T]$ represents the coarse registration matrix.

### 3.2 Accurate registration based on the ICP algorithm

**Figure 3:**
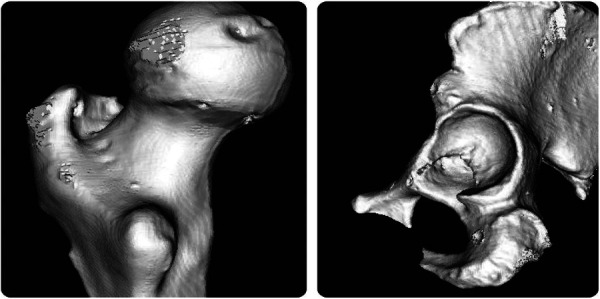
Femur (left) and acetabulum (right).

**Figure 4:**
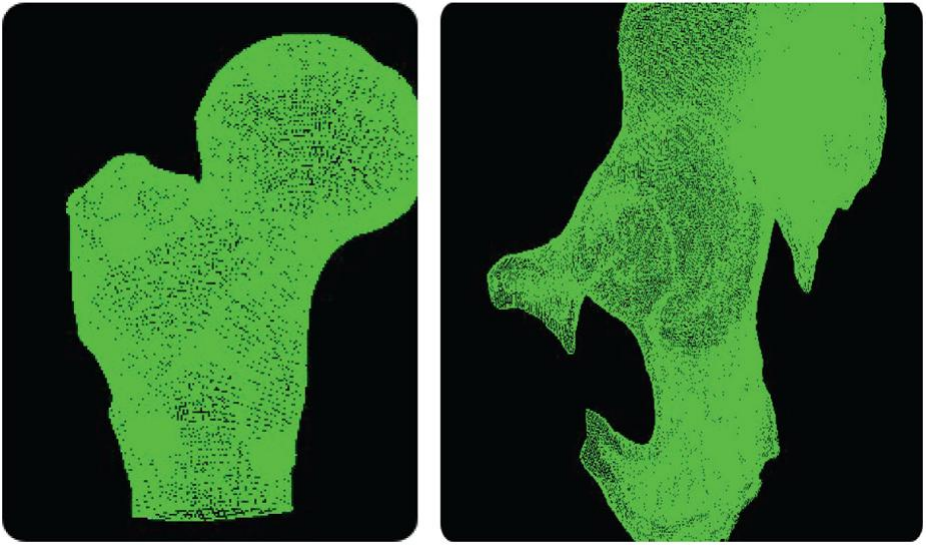
Femoral point cloud (left) and Acetabular point cloud (right).

Following coarse registration, an initial matrix transforming from the NDI to the CT coordinate system is established. A tracked probe is then used to sample points evenly on the hip joint and femoral head, as shown in Figure 3. Owing to the restricted incision of minimally invasive surgery, only 30 NDI points are acquired for fine registration. The CT point cloud is obtained by surface-rendering the CT volume and extracting its vertex coordinates, as shown in Figure 4.

The precise-registration procedure is as follows. The NDI point cloud comprises only 30 points, whereas the CT point cloud contains tens of thousands. For fine registration, the CT point cloud is restricted to the ipsilateral hip joint and femoral head within the target region.During surgery, cartilage covering the femoral head and acetabulum, together with variations in each surgeon’s probing technique, prevent the probe tip from consistently contacting the underlying bone surface.To mitigate the resulting error, fine registration must ensure that the majority of its selected points coincide. Therefore, this paper adopts a point-to-plane based sparse registration method, using the L2 distance and setting the penalty weight coefficient to 0.5. By switching from point-to-point to point-to-plane registration, the classical ICP objective is replaced, the registration problem is transformed into the form represented by Equation 10:

$$\frac{\arg\min}{R,T}=\sum_{i=1}^{N}L(n_{i}^{T}(Rx_{i}+T,Y))+I_{\mathrm{SO}(k)}(R),$$ (10)

where $|n_{i}^{T}(Rx_{i}+T-y_{i}){|}^{p}$ is the loss function, R is a 3×3 rotation matrix, T is a 3×1 translation vector, $x_{i}$ is the i-th point in the source point cloud, $y_{i}$ is the i-th point in the target point cloud, $n_{i}$ is the normal vector corresponding to the target point, Y is the set of point-to-plane residual vectors, $I_{\mathrm{SO}(k)}(R)$ is the rotation constraint indicator function, R∈SO(3); $I_{y}(y_{i})$ corresponds to the residual constraint, and N is the number of point pairs.

However, when intraoperative sampling points are limited and measurement errors exist, squared errors are sensitive to outliers. If erroneous matching occurs, the error becomes extremely large, causing the registration to be biased. Therefore, the $L_{p}$ sparse-induced norm is introduced, which automatically ignores large errors, and the weights of erroneous matches automatically decrease.

Where 0<p<1 is the sparsity-inducing parameter. Based on experimental validation, when p=0.5, the optimal balance between robustness and convergence stability is achieved; therefore, this parameter is adopted in this study.

Since Equation 10 is discontinuous and cannot be solved directly, it is transformed into alternating solutions of Equations 11, 12:

First step: Correspondence search with fixed R and T, optimizing Y, i.e., finding the closest points in the model point cloud. The expression is as follows:

$$\mathrm{Step}1\;:\;\frac{\arg\min}{Y}\sum_{i=1}^{N}{\left\|n_{i}^{T}(Rx_{i}+T-y_{i})\right\|}_{2}^{1/2}+I_{y}(y_{i}),$$ (11)

Second step: Rigid transformation optimization with fixed correspondences. The expression is as follows:

$$\mathrm{Step}2\;:\;\frac{\arg\min}{R,t}\sum_{i=1}^{N}{\left\|n_{i}^{T}(Rx_{i}+T-y_{i})\right\|}_{2}^{1/2}+I_{\mathrm{SO}(K)}(R).$$ (12)

Since it is a non-convex problem that cannot be solved analytically for the optimal solution, an auxiliary variable $z_{i}=n_{i}^{T}(Rx_{i}+T-y_{i})$ is introduced. Thus, the problem is transformed into $\underset{R,T,z}{\min}\sum_{i=1}^{N}{\left|z_{i}\right|}^{p}$. Decoupling the sparse regularization term and rigid body constraint edges, and defining the constrained residuals $\delta_{i}=n_{i}T(Rx_{i}+T-y_{i})-z_{i}$. Then construct the augmented Lagrangian function $L(R,T,z,\lambda )=\sum_{i=1}^{N}{\left|z_{i}\right|}^{p}+\sum_{i=1}^{N}\lambda_{i}\delta_{i}^{2}+\frac{\mu }{2}\sum_{i=1}^{N}\delta_{i}^{2}+I_{so(3)}(R)$, where $\lambda_{i}$ is the Lagrange multiplier, μ is the penalty parameter and $I_{\mathrm{so}(3)}(R)$ represents the rotation matrix constraint. In this paper, the penalty parameter μ is set to 1.0 and remains unchanged during the optimization process. The problem is solved using the alternating direction method of multipliers with R, T and λ fixed to solve for $z_{i}^{k+1}=\arg\underset{z_{i}}{\min}|z_{i}{|}^{p}+\frac{\mu }{2}(z_{i}-{\tilde{e}}_{i}{)}^{2}$, where ${\tilde{e}}_{i}=n_{i}^{T}(Rx_{i}+T-y_{i})+\frac{\lambda_{i}}{\mu }$. This problem is solved through threshold iteration. With z and λ fixed, optimizing $\underset{R,T}{\min}\sum_{i=1}^{N}{\left(n_{i}^{T}(Rx_{i}+T-y_{i})-z_{i}\right)}^{2}$, this problem is equivalent to the least squares problem of point-to-plane ICP.

Since the rotation matrix R is a nonlinear variable, it is linearized using small-angle approximation. Assuming the rotation vector is $\omega =(\omega x,\omega y,\omega z{)}^{T}$, the rotation matrix can be approximately expressed as $R\approx I+[\omega {]}_{\times }$, where $[\omega {]}_{\times }=\left[\begin{array}{ccc} 0 & -\omega_{z} & \omega_{y}\\ \omega_{z} & 0 & -\omega_{x}\\ -\omega_{y} & \omega_{x} & 0 \end{array}\right]$. Substituting it into the error function yields $r_{i}=n_{i}^{T}(x_{i}-y_{i})+n_{i}^{T}T+n_{i}^{T}(\omega \times x_{i})-z_{i}$. The unknown variables are expressed as $\xi =\left[\begin{array}{c} \omega \\ T \end{array}\right]$, $A_{i}=\left[\begin{array}{cc} {\left(x_{i}\times n_{i}\right)}^{T} & n_{i}^{T} \end{array}\right]$ and $b_{i}=z_{i}-n_{i}^{T}(x_{i}-y_{i})$. All stacked matrices are represented as e=Aξ−b, The least squares solution for $\underset{\xi }{\min}\|A\xi -b{\|}^{2}$ yields the normal equation $\left\{ \begin{array}{c} (A^{T}A)\xi =A^{T}b\\ \xi ={\left(A^{T}A\right)}^{-1}A^{T}b \end{array}\right.$. Solving the equation yields result $\xi =\left[\begin{array}{c} \omega \\ \Delta t \end{array}\right]$. Update $R\leftarrow \exp([\omega {]}_{\times })R$, T←T+Δt and $\lambda_{i}^{k+1}=\lambda_{i}^{k}+\mu (n_{i}^{T}(Rx_{i}+t-y_{i})-z_{i})$, the above process alternates with correspondence search until the registration error converges.

Next, the point cloud normal computation process is introduced. For each point in the target point cloud, the local normal vector is estimated using a plane fitting method based on principal component analysis (PCA). Let the neighborhood point set of $q_{i}$ be $N(q_{i})=\{x_{1},x_{2},\ldots ,x_{k}\}$, the number of neighborhood points is set to k=30 in our paper, and its centroid be $\bar{x}=\frac{1}{k}\sum_{\;j=1}^{k}x_{j}$. Then the covariance matrix of the neighborhood points can be expressed as: $C=\frac{1}{k}\sum_{\;j=1}^{k}(x_{j}-\bar{x}){\left(x_{j}-\bar{x}\right)}^{T}$. By performing eigenvalue decomposition on the covariance matrix, the eigenvalues and the corresponding eigenvectors $Cv_{j}=\lambda_{j}v_{j},j=1,2,3$ are obtained, satisfying $\lambda_{1}\leq \lambda_{2}\leq \lambda_{3}$. Since the local surface has the smallest dispersion in the direction of the normal, the eigenvector $v_{1}$ corresponding to the smallest eigenvalue $\lambda_{1}$ is taken as the local normal vector of the point, that is: $n_{i}=v_{1}$. This method is equivalent to local plane fitting in the least squares sense, with the objective of minimizing the sum of squared perpendicular distances from the neighborhood points to the fitted plane.

Corresponding point search adopts a nearest neighbor strategy, using a kd-tree for nearest neighbor search. For each source point, the closest point in the target point cloud is searched as a candidate corresponding point. To ensure matching robustness, filter the corresponding points using a distance threshold $d_{\max}=5.0\;\mathrm{mm}$, removing points that exceed the maximum allowable distance. The iteration stopping conditions are: (1) Maximum number of iterations $k_{\max}=100$; (2) Pose increment below a threshold ‖Δt‖<0.01mm, $\|\Delta \theta \|<0.01^{\circ }$; (3) Change in the sparse objective below a threshold $|E^{\left(k\right)}-E^{\left(k-1\right)}|<10^{-6}$.

Compared with traditional ICP, this method is robust to outliers; the sparse $L_{p}$ norm can automatically reduce the weights of points with large errors, making it suitable for sparse sampling scenarios. It can still achieve stable registration when only 30 anatomical points are collected intraoperatively.

### 3.3 Intraoperative surface feature point acquisition

In surgical navigation systems, due to the limited intraoperative workspace and time constraints, it is difficult to obtain a large number of bone surface points. Typically, only a small number of anatomical surface points can be collected on the bone surface using the NDI optical localization probe for registration.

During hip joint surgery, the surgical incision is small and the exposed bone surface area is limited; typically, only a small number of surface points from the medial and lateral regions of the acetabulum can be obtained. Meanwhile, to obtain stable spatial positioning points, the probe needs to penetrate the periosteum and contact the bone surface to complete the measurement. During the acquisition process, the probe needs to be kept stable to reduce positioning errors; therefore, the collection of a single point usually requires a certain amount of time.

If too many points are collected, the intraoperative operation time will be significantly prolonged, thereby increasing the surgical burden. In addition, during manual acquisition, it is difficult to ensure that the orientation and position of the probe contacting the bone surface are completely consistent each time, which may result in certain measurement errors in the collected points.

On the other hand, if the number of collected points is too small, it may lead to insufficient registration constraints, thereby affecting registration accuracy and the stability of the navigation system. Therefore, a trade-off among surgical time, operational feasibility, and registration accuracy is required when determining the number of intraoperative point acquisitions.

From a geometric constraint perspective, rigid body transformation has 6 degrees of freedom (3 rotations and 3 translations), and theoretically at least 3 non-collinear points are required to determine the rigid body transformation. However, in practical applications, if the number of collected points is too small, the registration result becomes highly sensitive to measurement noise. When the number of sampling points is between 3–10, the registration is usually extremely unstable, and with 10–20 points, it remains susceptible to noise influence.

Currently available commercial surgical navigation systems (such as the Stryker hip navigation system) typically collect approximately 32 surface points intraoperatively for registration, while in existing orthopedic surgical robot systems, the number of intraoperatively collected surface points is usually also above 30.

Based on the above clinical operational conditions and registration stability considerations, this study employs approximately 30 intraoperative sampling points for registration with the bone surface model reconstructed from preoperative CT, aiming to ensure registration accuracy while reducing intraoperative acquisition time.

## 4 A case study in registration experiments

### 4.1 Experimental configuration

To validate the effectiveness of the proposed registration algorithm, this study uses open-source CT data as experimental subjects and conducts registration experiments on hip joint and femoral models. First, the acetabulum and femur in the CT data are segmented and three-dimensionally reconstructed, and the reconstructed 3D models are then 3D printed. Subsequently, the 3D printed hip bone and femoral models are scanned again with CT, and 3D surface models and point cloud data are extracted from the scan data for subsequent registration experiment validation.

The registration algorithms, data processing, and graphical visualization involved in this study were all developed based on the C++ programming language, and implemented using Qt and VTK for interactive visualization programming. The experimental operating environment was the Windows 7 × 64 operating system, with a hardware platform consisting of an ® Core™ i7-12700K processor equipped with 16 GB of RAM.

The specific experimental procedure is as follows. First, the femur and acetabulum in the original CT data are segmented and surface-reconstructed. To ensure the geometric accuracy of the experimental models, Mimics software is used for segmentation and three-dimensional reconstruction when extracting the femoral and acetabular surface models, so as to obtain high-quality 3D surface models.

**Figure 5:**
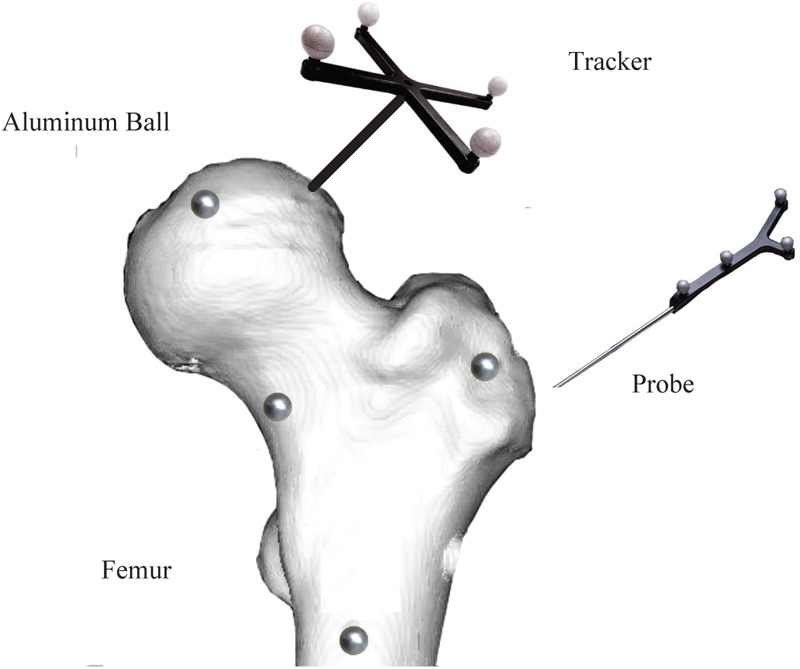
Femoral fixture schematic diagram.

**Figure 6:**
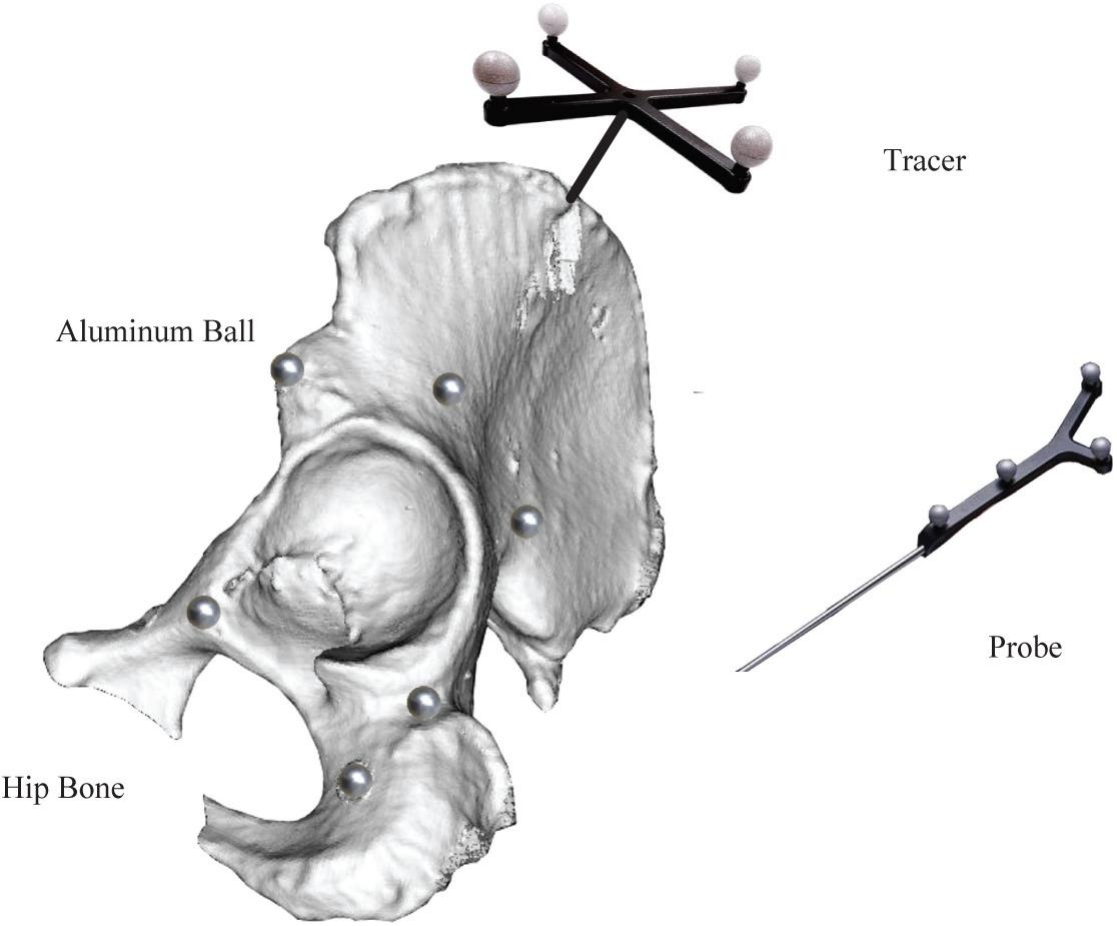
Acetabular fixture schematic diagram.

Subsequently, the reconstructed STL model files are 3D printed to obtain physical acetabular and femoral models. To construct the experimental coordinate system, the NDI optical tracker and femur are fixed at specific positions, and a femoral fixture is fabricated. Four aluminum marker balls are installed around the femur as spatial positioning reference points, as shown in Figure 5. The same method is used to fabricate the acetabular fixture, as shown in Figure 6.

After completing the fixture fabrication, the assembled fixture assembly is CT scanned, and surface models and point cloud data are extracted from the scan data. Meanwhile, a coordinate measuring machine (CMM) is used to measure the positions of the aluminum ball centers in the NDI tracker coordinate system. In addition, the 3D Blob analysis method is employed to segment the aluminum balls in the CT images and calculate their sphere centers, thereby obtaining the spatial coordinates of the aluminum balls in the CT coordinate system.

By using the known coordinates of the aluminum balls in the NDI tracker coordinate system and the coordinates of the aluminum balls in the CT coordinate system, the rigid body transformation relationship between the two coordinate systems can be calculated, thereby obtaining the transformation matrix between the NDI coordinate system and the CT coordinate system. This transformation matrix serves as the Ground Truth reference in the experiments for evaluating the accuracy of the registration algorithm.

Although human experiments have certain clinical significance, the use of 3D printed models in this study is based on the following considerations: First, to achieve high-precision quantitative validation, this study establishes a precise spatial reference system by pre-embedding aluminum marker balls in the femur and acetabulum, which cannot be performed in human subjects, making it impossible to obtain accurate ground truth for registration. Secondly, existing commercial software typically evaluates registration results by measuring the distance from the probe tip to the bone surface, but this method cannot reflect true errors when rotational errors exist. Using models with marker balls allows simultaneous verification of both translation and rotation accuracy, making the algorithm evaluation more comprehensive and reliable. Finally, conducting such accuracy validation on human subjects involves invasive procedures and requires strict medical ethics approval, whereas using printed models can avoid ethical risks while ensuring experimental reproducibility. In addition, model extraction and CT center point calculations during the experiments were all processed using medical-specific software to avoid interference from other errors.

In summary, our study employs 3D printed bone models for registration validation, which ensures the scientific rigor of precision testing while also considering experimental safety and reproducibility, providing a reliable basis for algorithm performance evaluation.

### 4.2 Analysis of the results

For algorithm validation, a tracked pointer was used to collect 30 points from the surgical surfaces of the femur and acetabulum. These points were evenly distributed around the rim of the acetabular cup and the femoral head resection plane. Comparison of the fine-registration results with the theoretical values. After fine registration, the aluminum-sphere coordinates in the NDI frame were transformed into the CT coordinate system. The residual error between each sphere’s centroid and its corresponding theoretical position was computed. This entire procedure was repeated 30 times, and the resulting errors were statistically analyzed.

To ensure fairness and reproducibility in the comparison between different registration algorithms, this paper adopts an identical experimental protocol for all competing methods. The detailed specifications are as follows.

1. The initialization is consistent. All algorithms use the same three-point coarse registration result as the initial pose for fine registration, and no method is allowed to use additional prior pose information or a better initialization approach. Therefore, any differences in subsequent registration performance among the methods arise solely from the fine registration algorithms themselves, rather than from differences in initialization conditions.
2. The region of interest (ROI) is consistent. All methods perform registration within the same local bone surface region of interest (ROI). This ROI is confined to the bone surface near the acetabular rim and the femoral head surgical area, based on the surgical exposure, ensuring that all algorithms carry out corresponding point search and error optimization within the same anatomical constraints.
3. The sampling points are consistent. In each repeated experiment, all algorithms use the exact same set of intraoperative probe sampling points as the source point cloud input. That is, the 30 bone surface points collected in each experiment are fully shared across the five algorithms. Therefore, there is no performance deviation among different algorithms caused by differences in input point sets.
4. The preprocessing is consistent. All algorithms construct the target point cloud based on the same preoperative CT data and follow an identical point cloud preprocessing pipeline.

Therefore, the only difference among the comparison methods in this paper lies in the registration models and optimization strategies employed during the fine registration stage, such as point-to-point or point-to-plane error metrics, the use of sparse constraints, and specific iterative update schemes. The above settings ensure that the experimental results can objectively reflect the performance differences of the algorithms themselves, thereby enhancing the credibility of the experimental conclusions.

The test results of the five algorithms in this paper are shown in Tables 1, 2. The following provides specific explanations of the RMSE, maximum error, standard deviation, and TRE metrics. Among them, TRE serves as the core metric for evaluating the spatial localization accuracy of target points after registration and is used as the primary basis for comparison throughout this paper.

1. The expression for root mean square error (RMSE) is $\mathrm{RMSE}=\sqrt{\frac{1}{N}\sum_{i=1}^{N}{\left\|x_{i}-{\tilde{x}}_{i}\right\|}^{2}}$. It is defined to measure the average deviation between corresponding points after registration, where $x_{i}$ and ${\tilde{x}}_{i}$ are the corresponding points of the registered model and the reference model, respectively, and N is the number of points. The smaller the value, the closer the registration result is to the real model, making it the most commonly used global error metric.
2. The expression for the maximum point-to-point distance is $\mathrm{MaxErr}=\underset{i}{\max}\|x_{i}-{\tilde{x}}_{i}\|$. It is defined as the maximum distance from a point to its corresponding reference point after registration, reflecting the most severe local deviation in the registration, which helps identify outliers and local mismatches.
3. The expression for standard deviation (STD) is $\mathrm{STD}=\sqrt{\frac{1}{n}\sum_{i=1}^{n}{\left(e_{i}-\bar{e}\right)}^{2}}$. It describes the stability of the error distribution, i.e., the fluctuation of the errors of all corresponding points relative to the mean error in each registration, where $e_{i}$ represents the error of the i-th correspondence, $\bar{e}$ is the mean value of the errors of all corresponding points. The smaller the STD, the more concentrated the distribution of registration errors, and the better the stability of the algorithm.
4. The target registration error (TRE), which is the Euclidean distance between the true target point position and the target point position after registration, is expressed as $\mathrm{TRE}=\frac{1}{m}\sum_{\;j=1}^{m}\sqrt{{\left(x-x^{\prime }\right)}^{2}+{\left(y-y^{\prime }\right)}^{2}+{\left(z-z^{\prime }\right)}^{2}}$. Where (x,y,z) represents the true spatial position of the aluminum sphere in the CT coordinate system, $\left(x^{\prime },y^{\prime },z^{\prime }\right)$ is the spatial coordinate of the aluminum sphere calculated through registration transformation, and m is the number of aluminum spheres. It reflects the final positioning error of the surgical navigation at the target point. A smaller TRE value indicates higher registration accuracy and smaller navigation system positioning error.

**Table 1:** Comparison of acetabular registration results.

Registration algorithms	TRE (mm)	RMSE (mm)	STD (mm)	MaxErr (mm)
Three-point coarse registration+ICP	3.76 ± 0.34	1.15 ± 0.22	1.08 ± 0.27	3.23 ± 0.31
Three-point coarse registration+FastICP	3.47 ± 0.31	1.23 ± 0.21	0.95 ± 0.25	3.5 ± 0.23
Three-point coarse registration+SparseICP	2.96 ± 0.2	0.78 ± 0.18	0.86 ± 0.19	2.47 ± 0.22
Three-point coarse registration+GICP	2.36 ± 0.23	0.57 ± 0.15	0.79 ± 0.21	2.86 ± 0.22
Three-point coarse registration+Ours	0.55 ± 0.15	0.3 ± 0.1	0.23 ± 0.09	1.24 ± 0.17

**Table 2:** Comparison of femoral registration results.

Registration algorithms	TRE (mm)	RMSE (mm)	STD (mm)	MaxErr (mm)
Three-point coarse registration+ICP	3.35 ± 0.32	1.56 ± 0.25	1.21 ± 0.23	3.76 ± 0.46
Three-point coarse registration+FastICP	3.15 ± 0.35	1.83 ± 0.27	1.05 ± 0.27	3.21 ± 0.43
Three-point coarse registration+SparseICP	2.87 ± 0.27	0.96 ± 0.16	0.93 ± 0.2	2.95 ± 0.28
Three-point coarse registration+GICP	2.57 ± 0.23	0.74 ± 0.18	0.85 ± 0.15	2.36 ± 0.24
Three-point coarse registration+Ours	0.67 ± 0.18	0.45 ± 0.12	0.19 ± 0.07	1.15 ± 0.19

Tables 1, 2 present the comparison of registration accuracy among different algorithms in femoral and acetabular experiments, respectively. It can be seen that the proposed algorithm achieves optimal results in terms of RMSE, maximum error, standard deviation, and TRE metrics. In the femoral experiment, TRE is reduced to 0.67 ± 0.18 mm, and in the acetabular experiment, TRE is reduced to 0.55 ± 0.15 mm, significantly outperforming traditional ICP, FastICP, SparseICP, and GICP algorithms. This demonstrates that the proposed method can achieve high-precision and high-stability registration effects even under conditions with a small number of sampling points.

To enhance the statistical rigor of the experimental results, this paper performed statistical analysis on the results of each algorithm across 30 repeated experiments. All continuous variables are presented as mean ± standard deviation (SD), and the bootstrap resampling method was used to estimate the 95% confidence interval (95% CI) for the difference in means.

Considering that the distribution of registration errors may not fully satisfy the normality assumption and that pairwise comparisons are made among algorithms under identical experimental conditions, this paper adopts the paired Wilcoxon signed-rank test to evaluate the significance of differences between the proposed method and each comparison algorithm in terms of the target registration error (TRE) metric, with a p<0.05 as the criterion for statistical significance.

Tables 3, 4 present the significance test results for the acetabular and femoral experiments, respectively. The results indicate that the proposed method demonstrates significant advantages over ICP, FastICP, SparseICP, and GICP in terms of TRE, with all *p*-values < 0.01, indicating that the performance improvements are not due to random fluctuations. Meanwhile, all 95% confidence intervals do not cross zero, further demonstrating that the proposed method achieves stable and consistent error reduction compared to existing methods.

**Table 3:** Significance test table for the acetabulum.

Comparison methods	Mean difference in TRE for the acetabulum (mm)	95% CI	*p*-Value
Ours vs ICP	−3.258	[−3.405,−3.110]	<0.01
Ours vs FastICP	−2.841	[−2.942,−2.739]	<0.01
Ours vs SparseICP	−2.436	[−2.510,−2.362]	<0.01
Ours vs GICP	−1.816	[−1.908,−1.725]	<0.01

**Table 4:** Significance test table for the femur.

Comparison methods	Mean difference in TRE for the femur (mm)	95% CI	*p*-Value
Ours vs ICP	−2.68	[−2.82,−2.54]	<0.01
Ours vs FastICP	−2.48	[−2.62,−2.34]	<0.01
Ours vs SparseICP	−2.20	[−2.31,−2.09]	<0.01
Ours vs GICP	−1.90	[−2.01,−1.7]	<0.01

To further validate the effectiveness of each component module of the method proposed in this paper, we designed ablation experiments and sequentially evaluated the following four registration configurations: coarse registration using only three points; point-to-point ICP introduced after coarse registration; point-to-plane registration (without sparse weighting) introduced after coarse registration; and the registration method used in this paper.

**Figure 7:**
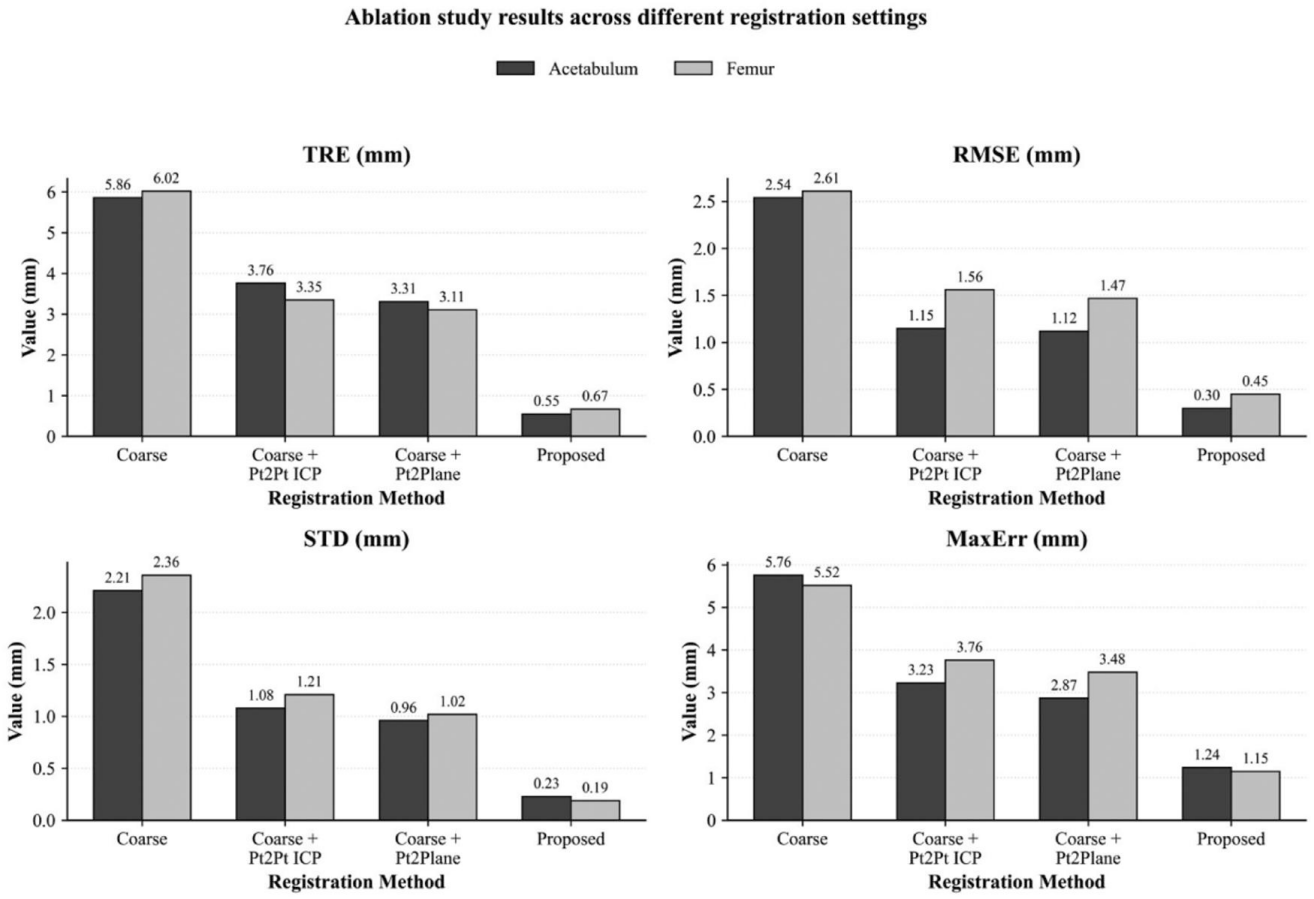
Ablation experiment results under different configurations of registration modules.

The results of the ablation experiments are shown in Figure 7. It can be observed that when using only three-point coarse registration, the TRE, RMSE, STD, and maximum error for both types of anatomical structures are relatively large, indicating that coarse registration can only provide an initial alignment and struggles to meet the requirements of high-precision navigation. After introducing point-to-point ICP on the basis of coarse registration, the overall error decreased significantly, indicating that local iterative optimization can effectively improve the accuracy of the initial registration. However, the TRE and maximum error remained relatively high, suggesting that the point-to-point error model has limitations in utilizing local geometric constraints on complex bone surfaces. After replacing the point-to-point error model with the point-to-plane model, the TRE, RMSE, and maximum error in both sets of experiments were further reduced, indicating that the point-to-plane constraint can more effectively utilize local surface normal information, thereby improving registration accuracy. Furthermore, after introducing sparse weighting on the basis of the point-to-plane model, the proposed registration method achieved the best results across all metrics, with particularly notable improvements in TRE and STD. This demonstrates that the sparse weighting mechanism can effectively suppress the influence of local outlier correspondences and gross errors, thereby further enhancing the robustness and stability of the registration. In summary, coarse registration, point-to-plane constraints, and sparse weighting all make clear contributions to the final performance improvement, and together constitute the key factors enabling the proposed method to achieve high-precision registration under sparse intraoperative point cloud conditions.

In this paper, we compared the performance of four configurations: coarse registration only, point-to-point ICP after coarse registration, point-to-plane registration (without sparse weighting) after coarse registration, and the proposed registration method. The results for the acetabulum and femur experiments are presented using four metrics: TRE, RMSE, STD, and maximum error. It can be observed that the proposed registration method achieves the best results across all metrics for both types of anatomical structures.

**Figure 8:**
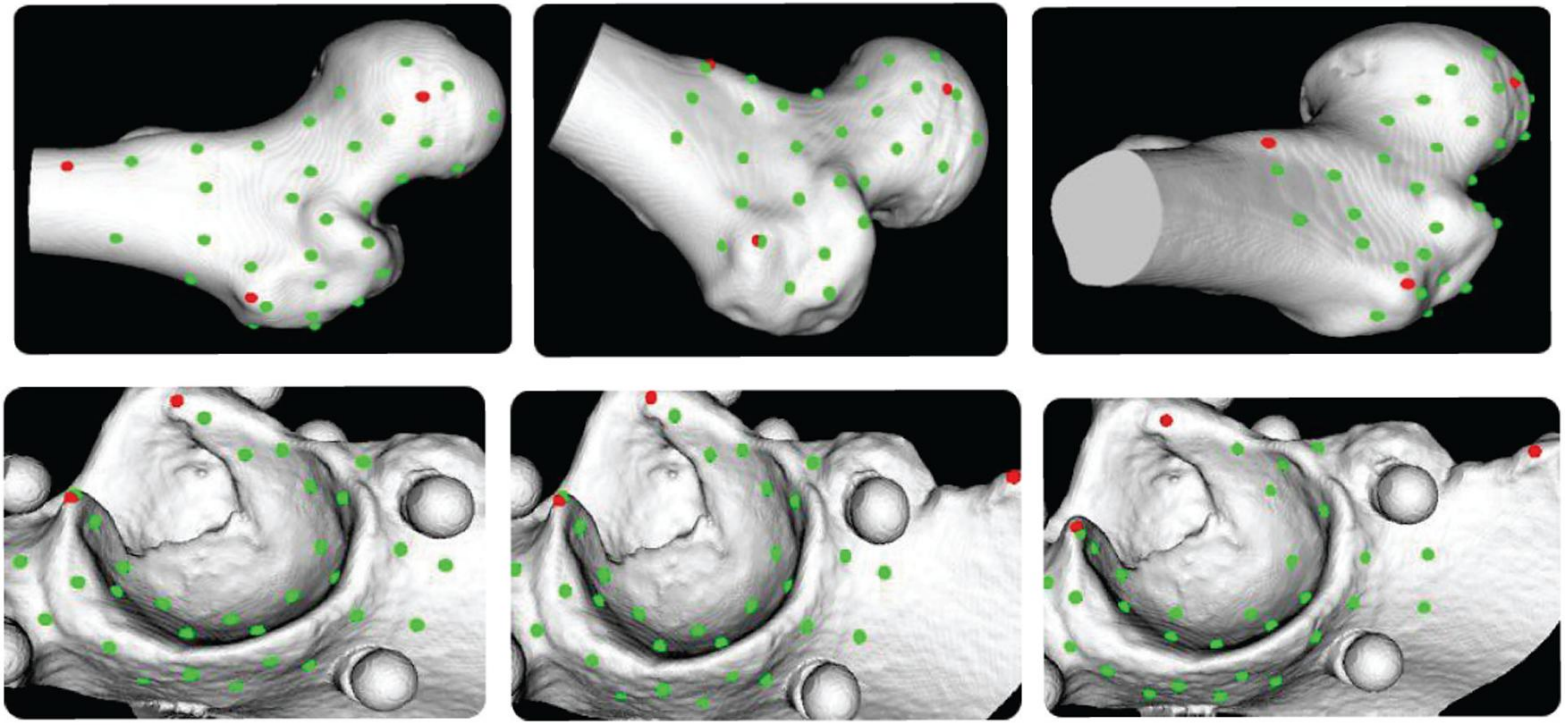
Registration of the acetabulum and femur.

Figure 8 shows the distribution of coarse- and fine-registration points on the bone surface after the complete registration workflow. Red points indicate the bone-surface landmarks selected for coarse registration, while green points mark those chosen for fine registration. The small spheres on the acetabular surface are aluminum balls embedded in the bone phantom for validating the actual registration accuracy. After registration, the bone-surface points acquired with the probe are evenly distributed and accurately coincide with the actual bone surface, showing neither penetration nor deviation, this result is consistent with the quantitative error analysis.

**Figure 9:**
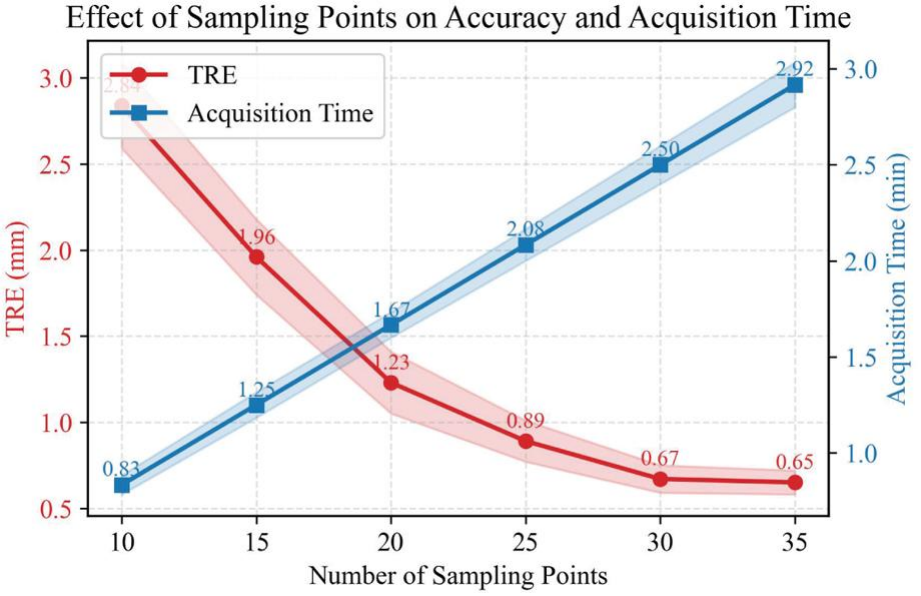
Influence of sampling point number on acetabular registration accuracy and acquisition time.

Figure 9 illustrates the influence of different sampling point quantities on acetabular registration accuracy (TRE) and sampling time. The results show that as the number of sampling points increases from 10 to 30, the average TRE decreases significantly from 2.84 mm to 0.67 mm, while the standard deviation also decreases, indicating that both registration accuracy and stability are improved. When the number of sampling points exceeds 30, the accuracy improvement tends to plateau, and sampling time increases linearly with the number of sampling points, from approximately 0.83 min for 10 points to 2.92 min for 30 points, and about 3 min for 35 points. Overall, there is a trade-off between accuracy and acquisition time: fewer sampling points allow for rapid acquisition but result in lower accuracy, while a larger number of sampling points can achieve higher accuracy but at the cost of increased time consumption. Considering both accuracy and operational efficiency, this study suggests that approximately 30 sampling points can achieve high-precision and stable acetabular registration within an acceptable acquisition time. If the registration verification time is included intraoperatively, the entire registration process can be controlled within 4 min.

The following is an additional note regarding this study. Although the registration method proposed in this paper achieves high accuracy and stability in experiments on 3D-printed acetabular and femoral models, the following limitations still exist in this study.

First, the validation objects in this study are 3D-printed bone models reconstructed and fabricated from CT data, rather than cadaveric specimens or in vivo subjects. Although 3D-printed models can faithfully reproduce bony anatomical structures and facilitate repeatable experiments and precise error evaluation under controlled conditions, they still cannot fully represent the complex scenarios encountered in real surgical environments.

Second, experiments on 3D-printed phantoms cannot fully simulate factors present in actual surgery, such as soft tissue occlusion, residual periosteum, limited intraoperative exposure, variations in probe contact posture, and individual patient anatomical differences. These factors may have additional effects on the quality of intraoperative point acquisition, the stability of correspondences, and the final registration accuracy in real total hip arthroplasty (THA). Therefore, the current experimental results primarily demonstrate that the proposed method achieves good geometric registration performance under controlled phantom conditions, but they cannot be directly equated to its final performance in real clinical scenarios. In future work, the robustness, reproducibility, and clinical applicability of this method need to be further validated through cadaveric experiments, intraoperative simulation experiments, and larger-scale clinical studies before stronger clinical conclusions can be supported.

## 5 Conclusion

This paper proposes a registration method for aligning intraoperative sparse point clouds with preoperative CT surface models in total hip arthroplasty navigation systems. By combining three-point coarse registration with an improved local sparse point-to-plane ICP fine registration, the proposed method achieves superior registration accuracy and stability compared to traditional ICP, FastICP, SparseICP, and GICP in experiments on 3D-printed acetabular and femoral models.

The experimental results show that under the condition of approximately 30 intraoperative sampling points, the proposed method can achieve high-precision local bone surface registration while maintaining operational efficiency and repeatability. This indicates that the method has good geometric registration performance and engineering feasibility under phantom experimental conditions.

It should be noted that the current study is still validated based on 3D-printed phantom experiments, and the results primarily support the technical feasibility of the method under controlled laboratory conditions. Future work is still needed to further validate its applicability and stability in real surgical scenarios through cadaveric experiments, intraoperative simulation studies, and clinical investigations.

## Data Availability

The raw data supporting the conclusions of this article will be made available by the authors, without undue reservation.

## References

[1] Subramanian P Wainwright TW Bahadori S Middleton RG. A review of the evolution of robotic-assisted total hip arthroplasty. Hip Int. (2019) 29:232–8. 10.1177/1120700019828286 30963802

[2] Nemati HM Christensson A Pettersson A Németh G Flivik G. Precision of cup positioning using a novel computed tomography based navigation system in total hip arthroplasty. Medicina (Kaunas). (2024) 60:1589. 10.3390/medicina60101589 39459376 PMC11509289

[3] Naito Y Sato T Nakamura Y. Registration in the supine position improves the accuracy of cup placement in total hip arthroplasty using a portable navigation system. Sci Rep. (2023) 13:1–9. 10.1038/s41598-023-47674-9 37980455 PMC10657446

[4] Scholes C Fatima M Schwagli T Liu D Ireland J. Imageless navigation system (Naviswiss) provides accurate component position in total hip arthroplasty with lateral decubitus position for end-stage hip osteoarthritis: a prospective cohort study with CT validation. Arthroplasty. (2024) 6:3. 10.1186/s42836-023-00224-0 38191491 PMC10773062

[5] Scholes C Schwagli T Ireland J. CT validation of intraoperative imageless navigation (Naviswiss) for component positioning accuracy in primary total hip arthroplasty in supine patient position: a prospective observational cohort study in a single-surgeon practice. Arthroplasty. (2023) 5:63. 10.1186/s42836-023-00217-z 38049889 PMC10696686

[6] Shimizu J Nagoya S Kosukegawa I Kanaizumi A Nakahashi N Teramoto A. The accuracy of cup placement in total hip arthroplasty (THA) using an augmented reality-based navigation system. Cureus. (2024) 16:e59432. 10.7759/cureus.59423 PMC11140827 38826595

[7] Tsukada S Ogawa H Hirasawa N Nishino M Aoyama H Kurosaka K. Augmented reality– vs accelerometer-based portable navigation system to improve the accuracy of acetabular cup placement during total hip arthroplasty in the lateral decubitus position. J Arthroplasty. (2022) 37:488–94. 10.1016/j.arth.2021.11.004 34763049

[8] Collyer J. Stereotactic navigation in oral and maxillofacial surger. Br J Oral Maxillofac Surg. (2010) 48:79–83. 10.1016/j.bjoms.2009.04.037 20061072

[9] Chen XJ Ye M Lin YP. Image guided oralimplantology and its application in the placement of zygoma implants. Comput Methods Programs Biomed. (2009) 93:162–73. 10.1016/j.cmpb.2008.09.002 18951648

[10] Maurer CR Fitzpatrick JM Wang MY Galloway RL Maciunas RJ Allen GS. Registration of head volume images using implantable fiducial markers. IEEE Trans Med Imaging. (1997) 16:447–62. 10.1109/42.611354 9263002

[11] Lin QY Yang R Cai K Si X Chen XW Wu XM. Real-time automatic registration in optical surgical navigation. Infrared Phys Technol. (2016) 76:375–85. 10.1016/j.infrared.2016.03.011

[12] Mijatović T Milovanović A Sedmak A. Integrity assessment of reverse engineered Ti-6Al-4V ELI total hip replacement implant. Struct Integrity Life. (2019) 19:237–42.

[13] Koide K Yokozuka M Oishi S Banno A. Voxelized gicp for fast and accurate 3D point cloud registration. In: *2021 IEEE International Conference on Robotics and Automation (ICRA)*. (2021). p. 11054–9.

[14] Le HM Do TT Hoang T Cheung NM. SDRSAC: semidefinite-based randomized approach for robust point cloud registration without correspondences. In: *2019 IEEE/CVF Conference on Computer Vision and Pattern Recognition (CVPR)*. (2019). p. 124–33.

[15] Yao ZW Zhao QX Li XF Bi QS. Point cloud registration algorithm based on curvature feature similarity. Measurement. (2021) 177:109274. 10.1016/j.measurement.2021.109274

[16] Shi XJ Liu T Han X. Improved iterative closest point(icp) 3D point cloud registration algorithm based on point cloud filtering and adaptive fireworks for coarse registration. Int J Remote Sens. (2020) 41:3197–220. 10.1080/01431161.2019.1701211

[17] Tan Y Zhu YC. Fireworks algorithm for optimization. Adv Swarm Intell pt 1 *Proc.* (2010) 6145:355–68. 10.1007/978-3-642-13495-1_44

[18] He Y Yang J Hou XM Pang SY Chen J. Icp registration with DCA descriptor for 3D point clouds. Opt Express. (2021) 29:20423–39. 10.1364/OE.425622 34266132

[19] Liu HB Liu T Li YP Xi MM Li T Wang YQ. Point cloud registration based on MCMC-SA ICP algorithm. IEEE Access. (2019) 7:73637–48. 10.1109/ACCESS.2019.2919989

[20] Arfat A Malik M Rab N Salman I Arshad S Javeria M Detection of omicron caused pneumonia from radiology images using convolution neural network (CNN). Comput Mater Contin. (2022) 74:3743–61. 10.32604/cmc.2023.033924

[21] Chen L Feng C Ma Y Zhao Y Wang C. A review of rigid point cloud registration based on deep learning. Front Neurorobot. (2024) 17:1281332. 10.3389/fnbot.2023.1281332 PMC10794353 38239758

[22] Deependra R Prashant J Sumit S Anand S Suman A Arfat Ahmad K, et al. Dual-stage deep learning framework for brain tumor classification and localization using multimodal MRI scans. Intell Based Med. (2026) 13:100361. 10.1016/j.ibmed.2026.100361

[23] Jia D Liu Y Zhang L. An efficient point cloud registration method based on deep learning framework. Geomatica. (2025) 77:100072. 10.1016/j.geomat.2025.100072

[24] Kujur A Raza Z Khan AA Wechtaisong C. Data complexity based evaluation of the model dependence of brain MRI images for classification of brain tumor and alzheimer’s disease. IEEE Access. (2022) 10:112117–33. 10.1109/ACCESS.2022.3216393

[25] Li Z Wang M. Rigid point cloud registration based on correspondence cloud for image-to-patient registration in image-guided surgery. Med Phys. (2024) 51:4554–66. 10.1002/mp.17243 38856158

[26] Liu Z Yang Z Jiang S Zhou Z. A spatial registration method based on point cloud and deep learning for augmented reality neurosurgical navigation. Int J Med Rob Comput Assist Surg. (2024) 20:e70030. 10.1002/rcs.70030 39716403

[27] Qin Z Yu H Wang C Guo Y Peng Y Ilic S, et al. Geotransformer: fast and robust point cloud registration with geometric transformer. IEEE Trans Pattern Anal Mach Intell. (2023) 45:9806–21. 10.1109/TPAMI.2023.3259038 37030771

[28] Thin M Arfat AK. Enhanced U-Net with attention mechanisms for improved feature representation in lung nodule segmentation. Curr Med Imaging. (2025) 21. 10.2174/0115734056386382250902064757 PMC13223473 40947694

[29] Zubair M Md Rais HB Ullah F Al-Tashi Q Faheem M Ahmad Khan A. Enabling predication of the deep learning algorithms for low-dose CT scan image denoising models: a systematic literature review. IEEE Access. (2024) 12:79025–50. 10.1109/ACCESS.2024.3407774

